# Functional characterization of CASP, a *CUX1* isoform, reveals its tumor-promoting role in colorectal cancer via TRIM21-mediated signaling

**DOI:** 10.1016/j.isci.2026.114783

**Published:** 2026-01-29

**Authors:** Biting Zhou, Wangxiong Hu, Wei Dai, Kailun Xu, Lihong Liu, Shu Zheng, Qichun Wei, Ting Chen

**Affiliations:** 1 Department of Radiation Oncology (Key Laboratory of Cancer Prevention and Intervention, China National Ministry of Education), The Second Affiliated Hospital, School of Medicine, Zhejiang University, Hangzhou, Zhejiang, China; 2 Cancer Institute (Key Laboratory of Cancer Prevention and Intervention, China National Ministry of Education), The Second Affiliated Hospital, School of Medicine, Zhejiang University, Hangzhou, Zhejiang, China; 3 Zhejiang Provincial Clinical Research Center for Cancer, Hangzhou, Zhejiang, China; 4 Cancer Center of Zhejiang University, Hangzhou, Zhejiang, China; 5 Department of Breast Surgery and Oncology (Key Laboratory of Cancer Prevention and Intervention, China National Ministry of Education), The Second Affiliated Hospital, School of Medicine, Zhejiang University, Hangzhou, Zhejiang, China

**Keywords:** Biological sciences, Molecular biology, Cell biology

## Abstract

CUT-like homeobox 1 (*CUX1*) is a transcription factor with dual roles in tumorigenesis. Among its splice variants, the Golgi-localized cut alternative spliced product (CASP) lacks DNA-binding domains, and its functional significance has remained largely unexplored. In this study, we identify CASP as a potential oncoprotein that is specifically upregulated in colorectal adenomas and carcinomas. Genetic silencing of CASP suppressed the proliferation and migration of colorectal cancer (CRC) cells, while its overexpression enhanced tumor growth and metastatic potential both *in vitro* and *in vivo*. Mechanistically, CASP interacts with the E3 ubiquitin ligase TRIM21 and promotes its ubiquitination and proteasomal degradation, leading to subsequent activation of the mitogen-activated protein kinase (MAPK) signaling pathway. Clinically, CASP expression was correlated with mismatch repair (MMR)/microsatellite instability (MSI) status and *TP53* mutational profiles. These findings implicate CASP in CRC progression and support its potential as a prognostic biomarker and therapeutic target by disrupting CASP-driven signaling.

## Highlights

• CASP (*CUX1* splice isoform) is dysregulated in colorectal cancer (CRC)
• CASP correlates with MMR/MSI status and *TP53* mutation status in CRC
• High CASP expression is associated with poor prognosis in CRC
• CASP binds TRIM21, promotes proteasomal degradation, and activates MAPK in CRC

## Graphical abstract

**Figure:**
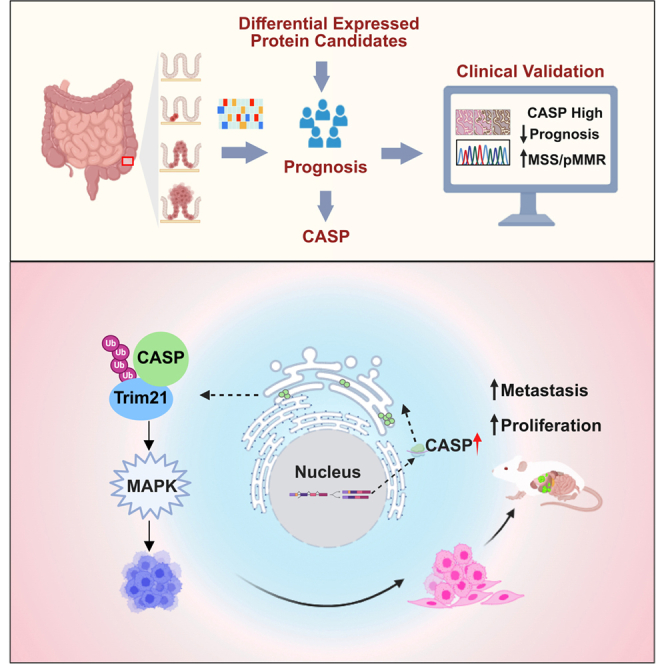

## Introduction

Colorectal cancer (CRC) is the third most common malignant tumor globally and ranks second in terms of mortality.^1^ It has been declining in high-income countries, largely due to effective screening programs while ascending especially in developed countries.^2^^,^^3^ Unlike other tumors, CRC is one of the few cancers that undergoes a malignant transformation from adenoma to carcinoma over a window of approximately 5–10 years. To identify candidate targets for understanding and disrupting the oncogenic processes, our previous study unveiled the protein signatures in tissue landscape of CRC evolving from normal colon to hyperplastic polyps, adenomas, adenocarcinoma not otherwise specified (AC), or mucinous adenocarcinoma (MC) by shotgun, and data-independent acquisition (DIA) mass.^4^ However, the analysis was limited in protein expression, without incorporating the data with prognostic factors or other relevant variables. Therefore, it is imperative to identify new biomarkers that with both ectopic expression and correlation with patients’ survival.

We re-investigated the expression mass data using updated version of DIA-NN software.^4^^,^^5^ Through mass resources and bioinformatic analysis in TCGA, cut alternative spliced product (CASP) was of particular interest among 21 identified proteins associating with patients’ prognosis. Moreover, unlike its well-known parental gene CUT-like homeobox 1 (*CUX1*), the protein product of this splice variant is an insufficiently studied protein, with its role barely characterized, and few studies reported to date.^6^^,^^7^

Before transcription and splicing to CASP, *CUX1*, the *Drosophila* cut gene mammalian homolog, was reported from the purification of the CCAAT-displacement protein (CDP) and has also been known as *CDP/CUT* or CUT-like1(*CUTL1*).^8^ *CUX1* is a ubiquitously expressed transcriptional factor that associated with cell proliferation, differentiation, migration, invasion, and DNA damage responses.^9^^,^^10^ Of note, *CUX1* has a paradoxical role both as a tumor suppressor and an oncogene depending on the isoforms under certain circumstances.^11^ These isoforms including a unique homeodomain and different repeats of DNA biding domain. The full-length mRNA generates a protein with a molecular weight of 200 kDa during translation (p200 CUX1), while the shorter mRNA generates a protein with a molecular weight of 75 kDa (p75 CUX1) during subsequent translation, and p200 CUX1 produces a protein with a molecular weight of 110 kDa (p110 CUX1) under the hydrolyzation of proteolytic enzyme L.^12^ However, the alternatively spliced product (CASP), which is spliced between exon 14 and 25 and ends at exon 33 is an exception (Figure S1). CASP just has a unique C-terminal region and an evolutional conserved region of coiled-coli.^7^ Without the four known DNA binding domains in *CUX1*, it was reported to be subcellularly localized in Golgi apparatus without known function.^6^ This small understudied protein aroused our interests in cancer.

This study identifies CASP, a Golgi-localized splice variant of *CUX1*, as being specifically upregulated in colorectal adenomas and carcinomas. Silencing of CASP inhibited the proliferation and migration of CRC cells, whereas expression of CASP significantly promoted the proliferation and migration of CRC cells *in vitro* and *in vivo*. Mechanistically, CASP was shown to negatively regulate the protein levels of the E3 ubiquitin ligase tripartite motif containing 21 (TRIM21) while positively modulating the MAPK pathway.^13^^,^^14^ Furthermore, clinicopathological analysis revealed a correlation between CASP expression and mismatch repair (MMR)/microsatellite instability (MSI) or *TP53* mutation functionality, with lower CASP expression observed in MMR-deficient or *TP53* wildtype samples. These findings highlight CASP as a potential oncogenic protein and suggest that targeting CASP-mediated tumor progression could be promising therapeutic strategies.

## Results

### Altered CASP expression in CRC patients

**Figure 1:**
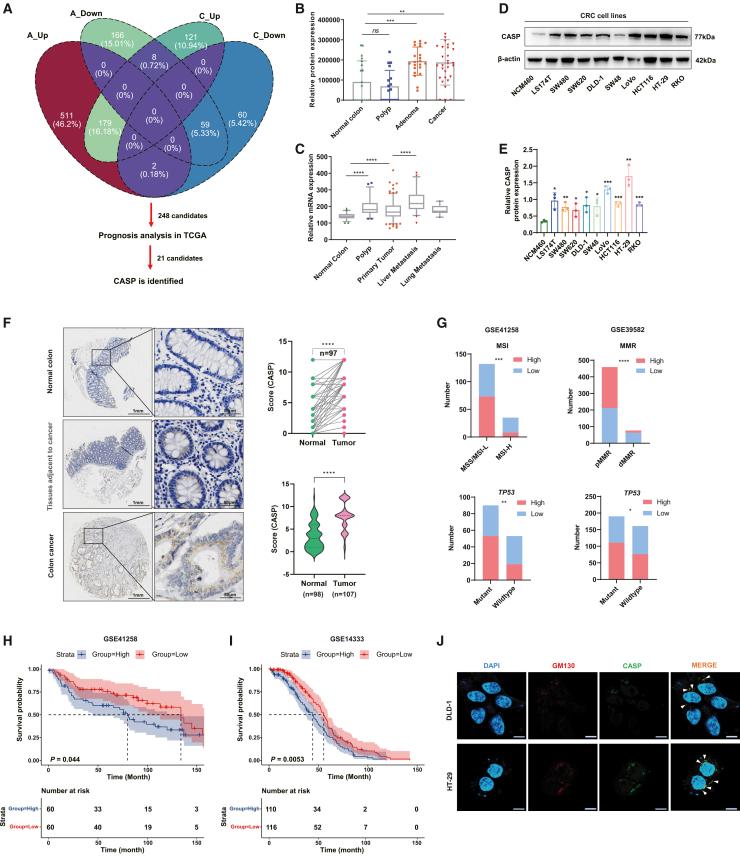
Altered CASP expression in CRC patients (A) Identification of CASP as a key prognostic marker via integrative analysis of expression profiles and TCGA survival data. Venn diagram showing the overlap of differentially expressed proteins between CRCs and normal tissues, as well as colorectal adenomas and normal tissues. A total of 248 candidates were analyzed for prognosis in TCGA, and 21 candidates were subsequently identified. (B) Protein expression of CASP in normal colon tissue (*n* = 20), polyps (*n* = 18), adenoma (*n* = 22), and CRC (*n* = 30) tissue based on MS proteomics data. (C) mRNA expression of CASP (probe 202367_at) in normal colon tissue (*n* = 54), polyps (*n* = 48), primary tumors (*n* = 185), CRC liver metastasis (*n* = 46), and CRC lung metastasis (*n* = 20) tissue from the GSE41258 dataset. (D) WB analysis of CASP expression in different CRC cell lines and the normal intestinal epithelial cell line NCM460. (E) The band intensities from WB analysis were quantified using ImageJ software and target protein bands was normalized to the corresponding control (β-actin or GAPDH) to account for variations in sample loading. (F) CASP expression was analyzed by IHC in 97 paired CRC/adjacent tissues (top right) and in 107 CRC samples plus 98 adjacent tissues (bottom right). Representative images are shown on the left. (G) Proportion of case distribution of MMR function/MSI status and mutations in *TP53* according to CASP expression in GSE41258 (left) dataset and GSE39582 (right) dataset. (H and I) Kaplan-Meier survival curves of CRC patients according to CASP expression using datasets from GSE41258 (*n* = 120) and GSE14333 (*n* = 226). (J) Co-staining of the Golgi marker GM130 and CASP in DLD-1 and HT-29 cells revealed clear evidence of colocalization in the Golgi apparatus; Scale bars: 10 μm. Data are expressed as mean ± standard deviation (SD). Statistical analyses were performed on GraphPad Prism. (B, C, E, and F- bottom right) Unpaired *t* test. (F- top right) Paired *t* test. (G) Chi-square test. ∗*p* < 0.05, ∗∗*p* < 0.01, ∗∗∗*p* < 0.001, ∗∗∗∗*p* < 0.0001.

Proteomic raw data acquired through DIA are permanently preserved, akin to fingerprints. As data analysis software or library advance, reanalyzing these datasets can yield additional insights. Using DIA-NN, we reanalyzed the proteomic profiles of formalin-fixed paraffin-embedded (FFPE) tissue samples from patients with hyperplastic polyps (*n* = 18), adenomas (*n* = 22), and CRC (*n* = 30), and normal negative margins (*n* = 20) as controls, from our previous study to identify differentially expressed proteins involved in cancer progression. By intersecting the differentially expressed proteins between normal tissues vs. adenomas (Figure S2A) and normal tissues vs. CRC (Figure S2B), we identified 248 overlapping proteins (Figure 1A).

To further investigate their potential roles in CRC, we evaluated the prognostic significance of their corresponding genes using the TCGA database, revealing 21 dysregulated genes significantly associated with prognosis. Notably, the *CUX1* gene attracted interest (Figure 1A). However, in our proteomic matrix, this gene corresponds to the alternatively spliced transcript CASP, which exhibited elevated expression in colon adenoma and cancer tissues (Figure 1B). To further investigate CASP expression in CRC, we analyzed its transcriptional levels based on the CASP-specific probe 202367_at in normal intestinal tissues (*n* = 54), polyps (*n* = 48), CRC tissues (*n* = 185), liver metastases (*n* = 46), and lung metastases (*n* = 20) from the GSE41258 dataset. CASP expression was significantly elevated in CRC tissues compared to normal tissues, with even higher expression in CRC liver metastases (Figure 1C). Subsequently, immunoblotting was conducted on six pairs of freshly frozen CRC tissues and their matched normal surgical margins, revealing a significant upregulation of CASP in CRC tissues compared to the paired normal tissues (Figures S2D and S2E). Similarly, CASP expression was markedly elevated in CRC cell lines compared to the normal intestinal epithelial cell line NCM460 (Figures 1D and 1E). Further immunohistochemistry (IHC) analysis of patient-derived samples (*n* = 108) and matched normal colon samples (*n* = 108) in two TMAs verified that CASP protein expression was significantly higher in CRC tissues than in normal tissues, after excluding samples with missing tissues (one tumor and 20 normal samples) (Figure 1F).

Moreover, based on IHC results, CRC samples with scores of 9 or 12 were categorized as the high-expression (*n* = 30), while those with scores of 0–4 were classified as the low-expression (*n* = 23). CASP exhibited a tendency for higher expression in the MMR-proficient group, with lower expression in the MMR-deficient group, which also exhibited poorer differentiation (Table 1). However, CASP expression was not significantly correlated with age, sex, tumor location, tumor size, TNM stage, mucus production, vascular invasion, or perineural invasion (Table 1). To further confirm the correlation between CASP expression, MSI status, and other clinical parameters, we analyzed datasets GSE41258 and GSE39582. The results consistently demonstrated reduced CASP expression in MMR-deficient samples across both datasets (Figure 1G, upper). Additionally, CASP expression was associated with *TP53* mutations, displaying higher levels in samples with mutated *TP53*. (Figure 1G, lower). Given the strong correlation between CASP and MSI/MMR status in the GSE41258 and GSE39582 datasets, we further explored the immune landscape by stratifying patients into CASP-high and CASP-low groups based on the CASP-specific probe (202367_at). Utilizing both xCell and CIBERSORT algorithms, we observed distinct immune infiltration patterns associated with CASP expression.

**Table 1:** Correlation between CASP levels in colon cancer patients and their clinicopathological characteristics

Clinicopathological features	Number	High expression *N* (%)	Low expression *N* (%)	*p* value
**Age**				

<60	16	9 (56.25)	7 (43.75)	0.9629
≥60	36	20 (55.56)	16 (44.44)	–

**Gender**				

Male	27	13 (48.15)	14 (51.85)	0.2056
Female	26	17 (65.38)	9 (34.62)	–

**Tumor size**				

≤5 cm	27	15 (55.56)	12 (44.44)	0.8753
>5 cm	26	15 (57.69)	11 (42.31)	–

**Tumor location**				

Left	20	9 (45.00)	11 (55.00)	0.3295
Right	26	17 (65.38)	9 (34.62)	–
Transverse	7	4 (57.14)	3 (42.85)	–

**TNM stage**				

I/II	32	20 (62.50)	12 (37.50)	0.285
III/IV	21	10 (47.62)	11 (52.38)	–

**Mucus production**				

Yes	9	5 (55.56)	4 (44.44)	0.9445
No	44	25 (56.82)	19 (43.18)	–

**Differentiation degree**				

Well-moderate	45	29 (64.44)	16 (35.56)	**0.0063∗∗**
Poor	8	1 (12.50)	7 (87.50)	–

**Vascular invasion**				

Positive	4	2 (50.00)	2 (50.00)	0.7817
Negative	49	28 (57.14)	21 (42.86)	–

**Perineural invasion**				

Positive	2	2 (100)	0 (0)	0.2068
Negative	51	28 (54.90)	23 (45.10)	–

**MMR status**				

Deficient	10	4 (40.00)	6 (60.00)	**0.0380∗**
Proficient	29	22 (75.86)	7 (24.14)	–

Statistical analysis was performed using Chi-square test.

Specifically, the CASP-low group exhibited significantly higher immune and microenvironment scores, as well as increased infiltration of M1 macrophages across both cohorts. Collectively, these analyses indicate that CASP downregulation is linked to an inflamed tumor microenvironment in CRC (Figure S3). To further clarify the association between CASP transcription and prognosis, we analyzed CASP-specific probe 202367_at in the GEO database (GSE41258 and GSE14333). High CASP expression was associated with shorter overall survival (Figure 1H) or disease-free survival (Figure 1I) in CRC patients. Therefore, our analyses indicate that ectopic CASP expression is associated with CRC and correlates with patient survival in certain cohorts.

### CASP promoted the proliferation of CRC both *in vitro* and *in vivo*

**Figure 2:**
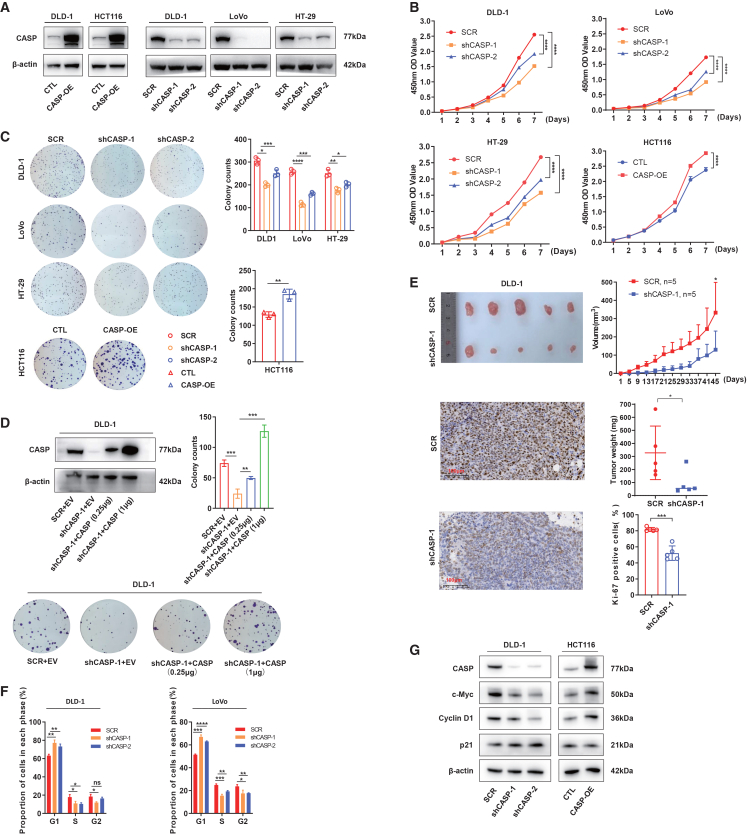
CASP promoted CRC cell proliferation both *in vitro and in vivo* (A) WB analysis of CASP protein level in control and CASP-OE DLD-1 and HCT116 cells (left) as well as scramble and CASP-KD DLD-1, LoVo, and HT-29 cells (right). (B) CCK-8 assay for investigating proliferation of the CASP knockdown in DLD-1, LoVo, and HT-29 cells, along with the CASP overexpression in HCT116 cells. (C) Colony formation ability of the CASP knockdown in DLD-1, LoVo, and HT-29 cells, along with the CASP overexpression in HCT116 cells; Quantification analysis is presented in the right histogram. (D) Colony formation rescue ability of CASP expression (shCASP-1 + CASP-OE) in DLD-1 cells; upper histogram represents quantification analysis. (E) Subcutaneous tumor formation in nude mice (*n* = 5/group) with CASP-KD cells; tumors derived from DLD-1 cells (left) and tumor growth curve of control (scramble, SCR) and CASP-KD DLD-1 cells in xenograft model (right IHC images of Ki-67 expression in xenograft tumor tissue and the result for Ki-67 positivity (%) is displayed in the lower panel. (F) The cell cycle distribution of CASP knockdown in DLD-1 and LoVo cells was analyzed by flow cytometry. (G) WB analysis of cell cycle related proteins was investigated. Data are expressed as mean ± SD. Statistical analyses were performed on GraphPad Prism. (B–F) Unpaired *t* test. ∗*p* < 0.05, ∗∗*p* < 0.01, ∗∗∗*p* < 0.001, ∗∗∗∗*p* < 0.0001.

To explore the biological function of CASP in CRC, we first examined its subcellular localization, confirming previous reports that it predominantly resided in the Golgi apparatus (Figure 1J). Then, we employed CRC cell line HCT116 for gain-of-function studies and DLD-1, LoVo, and HT-29 for loss-of-function studies according to baseline CASP levels (Figures 2A and S4A). Overexpression of CASP in HCT116 enhanced proliferation (Figure 2B) and increased both the number and size of colonies compared to the control (Figure 2C). Conversely, CASP inhibition in DLD-1, LoVo, and HT29 cells markedly reduced their colony-forming ability and proliferative capacity (Figures 2B and 2C). Of note, after reintroducing CASP expression, proliferation was restored and increased with elevated CASP levels in DLD-1 (Figure 2D).

We next investigated the role of CASP in CRC proliferation *in vivo* using a xenograft model with CASP-knockdown (CASP-KD) and control cells injected subcutaneously into nude mice. Consistent with *in vitro* results, DLD-1 (Figure 2E) and LoVo (Figure S4B) cells with low CASP expression exhibited reduced tumor growth rates. Tumor volumes in the CASP-KD group were significantly decreased compared to controls (Figure 2E, upper). Besides, the proportion of Ki67-positive cells was significantly lower in the CASP-KD group than in the scrambled control (Figure 2E, lower). Collectively, these results suggest that CASP promotes CRC proliferation both *in vitro* and *in vivo.*

To further elucidate the regulatory role of CASP in CRC proliferation, cell cycle analysis was performed in CASP-KD CRC cells. Flow cytometry revealed that CASP knockdown in DLD-1 and LoVo cells significantly increased the proportion of cells in the G1 phase while decreasing those in the S phase, indicating G1 phase arrest and inhibited cell proliferation (Figure 2F).

Further analysis using western blot (WB) showed that CASP knockdown led to downregulation of c-Myc and Cyclin D1, two key regulators of cell cycle progression, alongside an upregulation of p21, a cyclin-dependent kinase inhibitor. These molecular changes were aligned with the phenotypic effects observed due to CASP dysregulation on cell proliferation (Figure 2G). Thus, these findings collectively support that CASP regulates CRC cell proliferation by modulating the expression of essential cell cycle-related proteins.

### CASP promoted the metastasis of CRC both *in vitro* and *in vivo*

**Figure 3:**
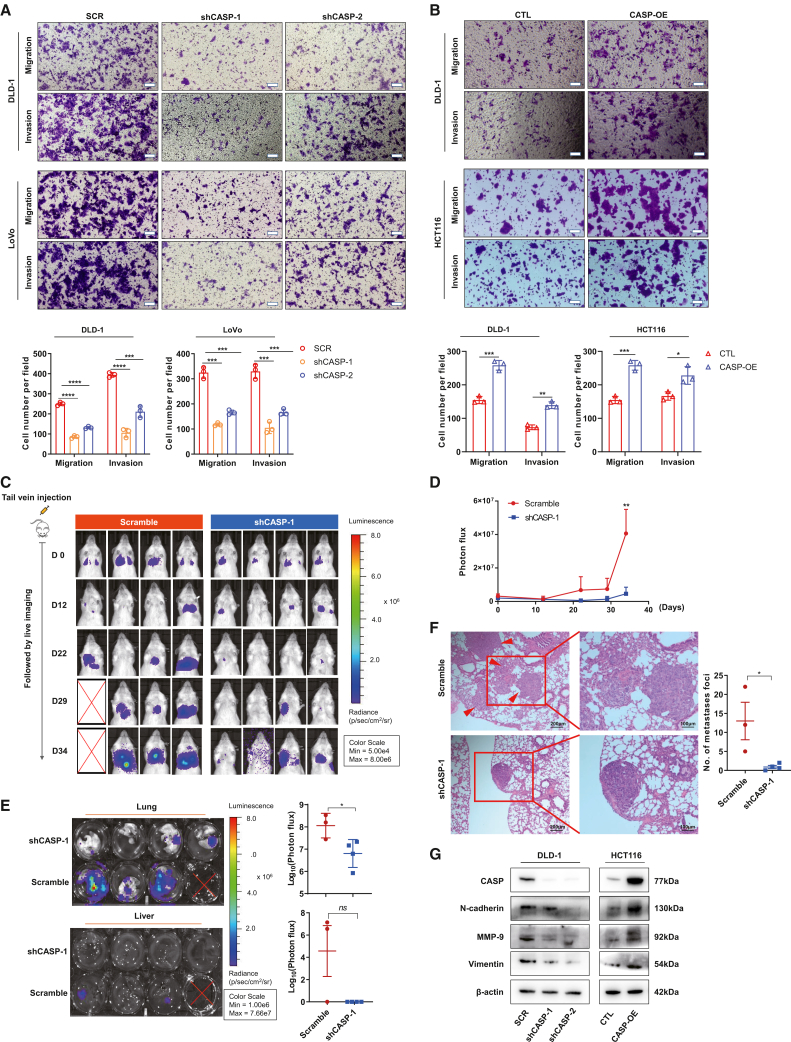
CASP promoted CRC cell metastasis both *in vitro and in vivo* (A and B) Transwell assay for investigating migration properties CASP knockdown (A) and overexpression (B) in DLD-1, LoVo and HCT116 cells; Scale bars: 100 μm. (C) Representative images of luciferase signals in metastatic foci formed by control and CASP-KD LoVo cells in immunodeficient mice. (D) Quantification of photon flux in metastatic foci formed by control and CASP-KD LoVo cells in immunodeficient mice. (E) Representative images and quantification of photon flux of the stripped liver and lung of from the sacrificed NSG mice in metastatic model. (F) H&E staining in pulmonary metastatic foci from the sacrificed NSG mice in metastatic model. (G) Changes in EMT-related protein markers and MMP-9 in response to CASP alteration in DLD-1 and HCT116 cells. Data are expressed as mean ± SD. Statistical analyses were performed on GraphPad Prism. (A, B, D, E, and F) Unpaired *t* test. ∗*p* < 0.05, ∗∗*p* < 0.01, ∗∗∗*p* < 0.001, ∗∗∗∗*p* < 0.0001, *ns* = no significant difference.

To investigate the role of CASP in CRC metastasis, we performed Transwell migration and invasion assays, which showed that CASP knockdown significantly, impaired the migratory and invasive abilities of DLD-1 and LoVo cells (Figure 3A). Conversely, CASP overexpression in DLD-1 and HCT116 cells markedly enhanced these capacities (Figure 3B).

We next examined CASP’s role in promoting CRC metastasis *in vivo* through tail vein injection of LoVo-shCASP-1 cells (stably expressing luciferase) into NSG immunodeficient mice, along with control cells. Bioluminescence imaging revealed reduced lung fluorescence in both groups on day 12. By day 22, fluorescence intensity in the control group significantly increased and surpassed that of the CASP-KD group, with a statistically significant difference observed by day 34 (*p* < 0.01) (Figures 3C and 3D). Upon euthanizing the mice, *ex vivo* fluorescence imaging of liver and lung tissues showed liver metastases in two mice from the control group, while none were detected in the CASP-KD group. In the lungs, the control group exhibited a 100% metastasis rate, while the CASP-KD group showed a reduced metastasis rate of 75%. Additionally, the fluorescence intensity of lung metastatic tumors in the control group was significantly higher than that in the CASP-KD group, consistent with the *in vivo* imaging results (Figure 3E). Hematoxylin and eosin (H&E) staining of lung tissue further confirmed that the number of metastatic foci in the CASP-KD group was significantly lower than in the control group (Figure 3F). Collectively, these findings indicate that CASP knockdown significantly suppresses the metastatic potential of CRC cells *in vivo*.

To corroborate these observations, WB analysis was performed to examine metastasis-associated molecules, focusing on epithelial-mesenchymal transition (EMT)-related and matrix metalloproteinase (MMP) proteins. CASP knockdown resulted in downregulated N-cadherin, Vimentin, and MMP-9, while CASP overexpression led to upregulation (Figure 3G). Taken together, these results underscore the crucial role of CASP in CRC metastasis.

### CASP promoted MAPK signaling pathways in CRC cells

**Figure 4:**
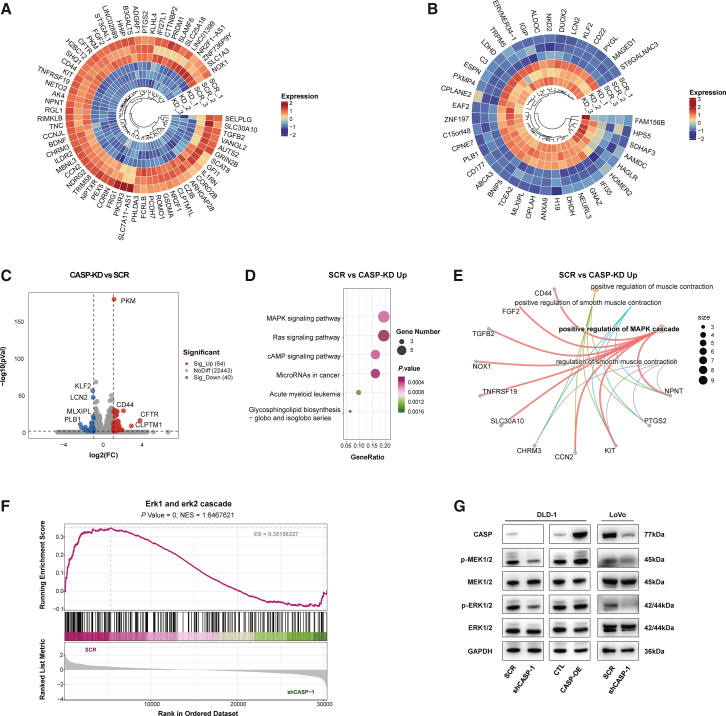
CASP promoted MAPK signaling pathways in CRC cells (A and B) Heatmaps for genes whose transcription were negatively related to CASP knockdown (A) and genes whose transcription were positively related to CASP knockdown (B), respectively (*p* < 0.05). Color bar: relative expression value. (C) Volcano map of differential genes in control and CASP-KD groups. Cutoff: Fold change >2, *p* < 0.05. (D) KEGG pathway analysis shows the significantly affected pathways upon CASP knockdown in DLD-1 cells. (E) GO biofunction enrichment plot shows the upregulated genes in the control group compared to the CASP-KD group. (F) GSEA analysis shows the aberrant regulation of ERK cascade. (G) The expression of phosphorylated MEK1/2 and ERK1/2 was detected by WB in CASP-altered cells in DLD-1 and LoVo.

To further elucidate the mechanisms by which CASP promotes CRC progression, RNA sequencing was performed on CASP-KD DLD-1 cells and their control counterparts. The analysis identified 64 upregulated genes negatively related to CASP knockdown (Figure 4A) and 40 downregulated genes positively related to CASP knockdown (Figure 4B). A volcano plot displayed the total differentially expressed genes between the control and CASP-KD groups, using a cutoff of fold change > 2 and *p* < 0.05 (Figure 4C). Among the upregulated genes, *CD44* and *PKM* emerged as points of interest. *CD44*, a well-known cancer stem cell marker, is involved in multiple critical pathways for stem cell self-renewal, tumor proliferation, EMT, and invasion. *PKM*, particularly the *PKM2* isoform, is crucial for cell proliferation. WB analysis confirmed that CASP knockdown in DLD-1 cells significantly reduced the protein levels of CD44 and PKM2, aligning with the RNA sequencing results (Figure S5F).

KEGG pathway enrichment analysis of the upregulated genes revealed that CASP primarily influences the MAPK signaling pathways, as well as cancer-related pathways (Figure 4D). Gene ontology (GO) functional enrichment analysis further highlighted CASP’s positive regulation of the MAPK cascade, a pathway critically involved in CRC progression (Figure 4E). Furthermore, gene set enrichment analysis (GSEA) suggested that CASP activates MAPK signaling pathways in CRC cells (Figure 4F). WB analysis in DLD-1 and LoVo cells confirmed that CASP levels positively correlate with MAPK pathway activation. Specifically, CASP knockdown suppressed the phosphorylation of MEK1/2 and ERK1/2, whereas CASP overexpression resulted in their upregulation (Figure 4G). Furthermore, CASP may affect the Wnt signaling pathway (Figure S5C) since the key molecule β-catenin was upregulated in CASP-OE DLD-1 cells via WB and immunofluorescence, while it was reduced in CASP-KD DLD-1 cells (Figures S5D and S5E).

In addition, to explore the effect of varying CASP expression on CRC at the protein level, 30 cases of CRC in MS data were equally divided into high-expression group and low-expression group. GO functional enrichment analysis of the DEPs showed that Golgi vesicle transport and glycosylation were predominantly enriched in proteins upregulated in the high-expression group (Figure S5A), while cell-substrate adhesion and extracellular matrix organization were mainly enriched in proteins upregulated in the low-expression group (Figure S5B).

### CASP impedes MAPK signaling by negatively regulating TRIM21 protein stability

To further elucidate the molecular mechanisms by which CASP influences CRC, Co-immunoprecipitation (Co-IP), and MS were employed to identify CASP-interacting proteins. The identified proteins underwent GO and KEGG functional and signaling pathway enrichment analyses, revealing that CASP-interacting proteins are primarily involved in RNA splicing and mRNA processing (Figure S6A). Consistently, the spliceosome pathway emerged as an enriched pathway (Figure S6B). Given that CASP is a variable spliceosome, the mRNA splicing-related proteins identified are likely upstream regulatory molecules. Nevertheless, uncovering the downstream regulatory interacting proteins is crucial for understanding CASP’s pro-carcinogenic role. Among the top 10 proteins with the highest interaction scores, TRIM21 was noted as a protein of particular interest. TRIM21 is a dual-localized protein found in both the cytoplasm and nucleus, characterized by an N-terminal RING domain with E3 ubiquitin ligase activity and a coiled-coil domain that mediates ubiquitination and protein degradation.^15^^,^^16^

**Figure 5:**
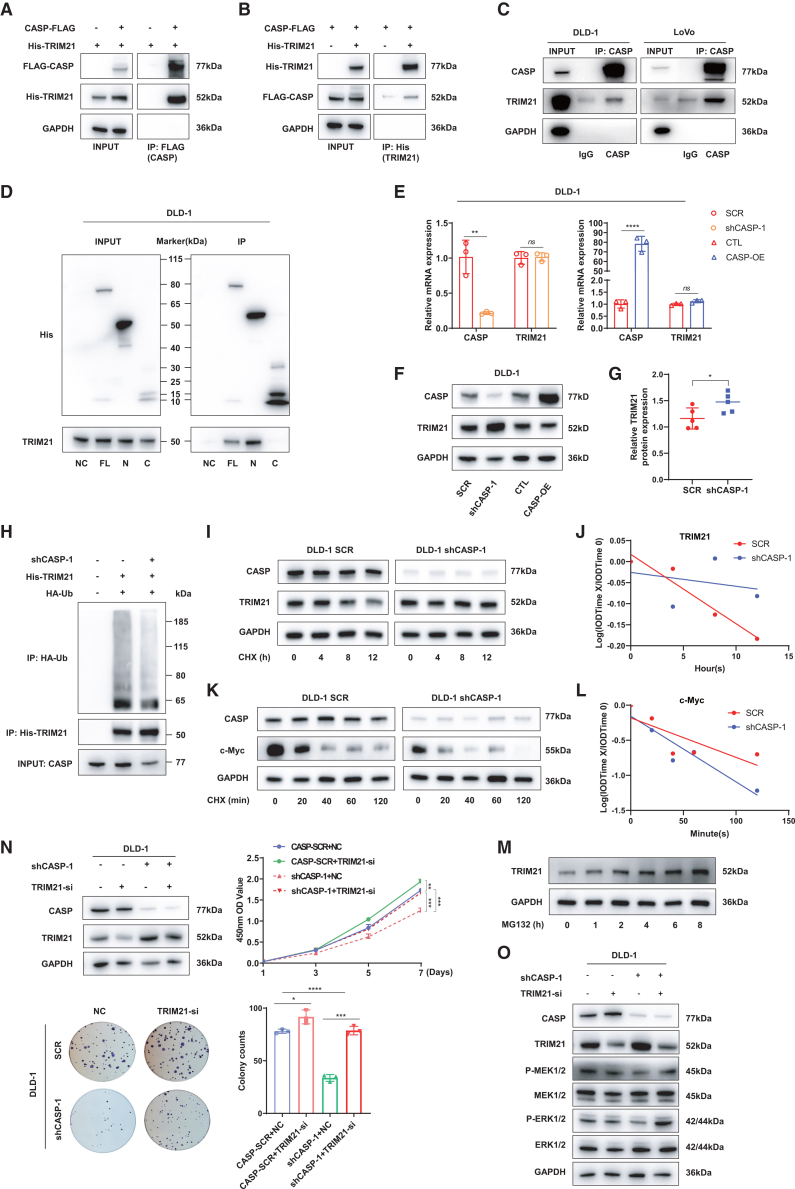
CASP impedes MAPK signaling by negatively regulating TRIM21 protein stability (A) Immunoblot analysis of TRIM21 immunoprecipitated using an FLAG tag fused with CASP in 293T cells. (B) Immunoblot analysis of CASP immunoprecipitated using a His tag fused with TRIM21 in 293T cells. (C) Immunoblot validation of endogenous co-precipitation of TRIM21 with CASP antibody in DLD-1and LoVo cells. (D) Co-IP analysis with an anti-His antibody was performed in DLD-1 cells 48h after transfection with constructs expressing the His-tagged full-length, N-terminal cytoplasmic, or C-terminal Golgi domain of CASP (or an empty vector), to assess their respective interactions with TRIM21. (E and F) qPCR (E) and WB (F) analyses of TRIM21 expression at the mRNA and protein levels, respectively, in DLD-1 cells following CASP knockdown or overexpression compared to their respective controls. (G) Quantification of TRIM21 protein expression from the WB shown (Figure S6C), which displays TRIM21 and CASP levels in protein extracts from subcutaneous xenograft tumors of nude mice. Band intensities for TRIM21 were normalized to β-actin (loading control) using ImageJ software. (H) 293T cells were co-transfected with plasmids encoding HA-Ub, His-TRIM21, and control or shCASP-1. Cells were treated with MG132 for 6 h prior to lysis. His-TRIM21 was immunoprecipitated from cell lysates using Ni-NTA beads, and its ubiquitination level was detected by immunoblotting with anti-HA antibody. (I and J) Turnover of TRIM21 protein was assessed in scrambled and CASP-KD DLD-1 cells treated with 60 μg/mL CHX for the indicated times. Representative blots (I) and quantification (J) demonstrating that CASP depletion significantly slows the degradation rate of TRIM21. (K and L) c-Myc turnover was analyzed in parallel under the similar conditions. Representative blots (K) and quantification (L) revealing that CASP knockdown accelerates the degradation kinetics of c-Myc. Protein levels were normalized to GAPDH and are presented as the percentage remaining relative to time 0. (M) DLD-1 cells were treated with the proteasome inhibitor MG132 (20 μM) for the indicated times (0, 1, 2, 4, 6, and 8 h). WB analysis was performed to monitor the stabilization of TRIM21 protein. (N) Alterations in the proliferative capacity of CASP-KD DLD-1 cells and their control counterparts after transient transfection with TRIM21 siRNA. Immunoblot analysis showing the knockdown efficiency of TRIM21(up-left); Cell viability measured on days 1, 3, 5, and 7 using the CCK-8 assay(up-right); Schematic representation of the cell colony formation assay (down-left); Statistical graph showing the number of colonies for each group (down-right). (O) Alterations in key molecules of the MAPK pathway in control and CASP-KD DLD-1 cells following TRIM21 downregulation. Data are expressed as mean ± SD. Statistical analyses were performed on GraphPad Prism. (E, G, and N) Unpaired *t* test. ∗*p* < 0.05, ∗∗*p* < 0.01, ∗∗∗*p* < 0.001, ∗∗∗∗*p* < 0.0001, *ns* = no significant difference.

Co-IP experiments in 293T cells with exogenous expression of TRIM21 and CASP (Figures 5A and 5B), as well as endogenous Co-IP experiments in CRC cell lines (DLD-1 and LoVo) (Figure 5C) confirmed their interaction. To identify the functional binding region of CASP, we tested various truncations of its full-length domains. In DLD-1 cells, we found that the N-terminal cytoplasmic topological domain of CASP interacted with TRIM21, whereas the C-terminal Golgi topological domain did not (Figure 5D). We further examined the regulatory crosstalk between CASP and TRIM21. Our data indicate that modulation of CASP levels negatively regulates TRIM21 protein abundance without altering its mRNA expression (Figures 5E and 5F), and this observation was further confirmed in xenograft tumor tissues (Figures 5G and S6C). At the mechanistic level, Co-IP assays showed that CASP depletion markedly reduced TRIM21 polyubiquitination (Figure 5H). In agreement with this, cycloheximide (CHX) chase assays demonstrated that CASP knockdown significantly slowed the turnover of endogenous TRIM21 (Figures 5I and 5J) while concomitantly accelerating the degradation of c-Myc, a key TRIM21 substrate^16^ (Figures 5K and 5L). Furthermore, treatment with the proteasome inhibitor MG132 led to a time-dependent accumulation of TRIM21 protein (Figure 5M), indicating that TRIM21 is predominantly removed via proteasome-dependent degradation. Taken together, these findings indicate that CASP promotes the proteasomal degradation of TRIM21 by facilitating its ubiquitination.

Collectively, our findings demonstrate that CASP interacts with TRIM21 and functions as its upstream regulator, targeting TRIM21 for ubiquitin-mediated proteasomal degradation and providing a foundation for future studies on the role of CASP in CRC progression.

To determine whether CASP exerts its pro-tumorigenic effects in CRC by regulating TRIM21, we transiently transfected TRIM21 siRNA into CASP-KD DLD-1 cells and their control counterparts. CCK-8 and colony formation assays revealed that TRIM21 downregulation restored the proliferation capacity of CRC cells suppressed by CASP knockdown (Figure 5N), suggesting that CASP may regulate CRC cell proliferation through TRIM21. We then examined whether CASP activates the MAPK signaling pathway via TRIM21. WB analysis showed that CASP knockdown alone significantly reduced the expression of phosphorylated MEK1/2 (*p*-MEK1/2) and ERK1/2 (*p*-ERK1/2). However, TRIM21 inhibition slightly reduced *p*-MEK1/2 and *p*-ERK1/2 expression in CASP-expressing cells, while in CASP-KD cells, *p*-MEK1/2 and *p*-ERK1/2 expression were markedly restored (Figure 5O).

These findings suggest that CASP activates the MAPK signaling pathway by interacting with and negatively regulating TRIM21, ultimately promoting its ubiquitin-mediated degradation.

## Discussion

The human *CUX1* gene is known to be transcribed into multiple isoforms that are evolutionarily and functionally conserved.^17^ Based on subcellular localization, these isoforms can be divided into two types: nuclear-localized CDP and Golgi-localized CASP.^7^ Nuclear-localized transcripts terminate at exon 24 of the gene and can be further spliced or hydrolyzed into different isoforms, often containing cut repeats and homology domains. In contrast, Golgi-localized isoforms are transcriptionally extended to distal exons, ultimately including exons 1–14 and 25–33 through selective splicing.

The nuclear-localized products have been extensively studied, revealing that *CUX1* undergoes somatic point mutations and loss of heterozygosity, leading to functional inactivation in several cancers. Since no mutations are found in the remaining alleles, *CUX1* appears to function as a haplo-insufficient tumor suppressor gene.^18^^,^^19^^,^^20^ Evidence suggests that reduced *CUX1* expression promotes tumorigenesis, whereas increased expression facilitates tumor progression.^21^^,^^22^ However, the Golgi-localized isoform, which is lack of the DNA-binding motifs and other functional domains of *CUX1*, and retains only the conserved coiled-coil portion remain understudied. Previous pioneering research by the Sean Munro’s group demonstrated that CASP was localized to the Golgi apparatus rather than the nucleus, as previously reported, and seemed more evolutionarily ancient than CDP because only the former was present in plants and fungi. Although the function of CASP was not elucidated, it seems likely to be related to that of giantin and golgin-84 because the proteins share an overall structure and conserved transmembrane domain residues. Studies on the homolog COY1 in *Saccharomyces cerevisiae* suggest that the transmembrane region of this type of Golgi-localized coiled-coil protein is involved in its function.^6^

Our study focused on the CASP of the *CUX1* gene, using proteomic data obtained from FFPE samples of normal, adenoma, and carcinoma tissues via DIA/MS combined with publicly databases. It was found that CASP is ectopically expressed in adenomas and carcinomas, which suggest CASP could serve as a biomarker for early-stage CRC screening. Given its unusual and evolutionarily conserved gene structure, CASP may have Golgi-specific functions independent of CDP. Using CRC as a model, we explored the potential role of CASP in tumorigenesis. Our analysis revealed that CASP expression was significantly elevated in CRC tissues compared to adjacent normal tissues and was associated with poor prognosis. Correlation analysis showed that CASP expression was related to MMR functionality and *TP53* mutation, with lower expression observed in MMR-deficient or *TP53* wildtype samples. Immune infiltration analyses suggested that low CASP expression was associated with a more inflamed tumor microenvironment in CRC, characterized by higher immune and microenvironment scores and increased infiltration of multiple immune cell subsets, particularly M1 macrophages, indicating that CASP downregulation is linked to an immunologically “hot” tumor microenvironment in CRC, which may have important implications for predicting or understanding responses to immunotherapy. Functionally, upregulation of CASP expression promoted the G1-to-S phase transition, enhancing cell proliferation and colony formation. Furthermore, CASP expression increased tumor cell motility and metastasis by influencing EMT and MMP related molecules. Mechanistically, CASP negatively regulates TRIM21 protein expression by promoting its ubiquitin-mediated degradation via a direct N-terminal interaction, which in turn influences the MAPK pathway and promotes CRC progression.

TRIM21 is a member of the TRIM protein family, which includes over 80 proteins characterized by conserved domains such as an N-terminal RING domain with E3 ubiquitin ligase activity, a B-box domain, and a coiled-coil domain.^15^ TRIM21 has been implicated in the regulation of inflammation, autoimmunity, and cancer.^23^^,^^24^^,^^25^ In this study, we identify CASP as an upstream regulator of TRIM21, revealing a previously uncharacterized interaction between the two proteins. We demonstrate that CASP promotes TRIM21 ubiquitin-mediated degradation, thereby negatively regulating TRIM21 protein stability. Collectively, our findings establish a molecular mechanism that is dysregulated during CRC progression. It is known that TRIM21 exhibits dual roles in cancer, functioning as either a tumor promoter or suppressor depending on the biological roles of its ubiquitination substrates.^26^^,^^27^ In this study, siRNA-mediated knockdown of TRIM21 enhanced the proliferation of CRC cells, suggesting that TRIM21 acts as a tumor suppressor in CRC. Furthermore, the reduced proliferation of CRC cells caused by CASP knockdown was reversed when TRIM21 expression was suppressed, indicating that CASP may promote CRC cell proliferation by regulating TRIM21.

In this study, WB analysis revealed that, in CASP-KD CRC cells, TRIM21 downregulation restored *p*-ERK1/2 expression, suggesting that CASP may activate the MAPK pathway by negatively regulating TRIM21. However, Ye et al. report that TRIM21 is a target of ERK2 in CRC, and that ERK2 phosphorylates TRIM21, thereby disrupting its interaction with c-Myc and preventing c-Myc degradation, suggesting that MAPK signaling is upstream of TRIM21,^16^ which appears inconsistent with our findings. Nevertheless, we propose that our results are not mutually exclusive with those of Ye et al. but rather complementary, collectively pointing to a complex feedforward/feedback loop regulating MAPK signaling. Integrating Ye et al.’s findings regarding ERK2-mediated phosphorylation, we propose a unified model: CASP acts as an upstream stability regulator, functioning primarily during pathological states or early signal activation. Elevated CASP levels appear to deplete TRIM21, creating a permissive environment for MAPK activation. Subsequently, activated ERK2 may reciprocally phosphorylate the remaining TRIM21, finely tuning its E3 ligase activity toward substrates such as c-Myc, which exhibits a rapid degradation rate upon CASP knockdown. Notably, while CASP knockdown significantly suppresses pathway activity, the activation effect of CASP overexpression on *p*-ERK1/2 appears relatively moderate. We interpret this observation as being consistent with a negative feedback mechanism: once CASP triggers MAPK activation, ERK-TRIM21-dependent regulation likely limits excessive signal amplification, providing collateral support for this bidirectional network. Additionally, the regulatory role of TRIM21 in the MAPK pathway has been partially characterized in previous studies. For example, HTR1A interacts with TRIM21 and PSMD7 to promote the ubiquitin-proteasome degradation of TGFβRII, thereby suppressing both the canonical Smad pathway and the non-canonical MEK/ERK/c-Myc pathway of TGFβ. This inhibition prevents cytoskeletal rearrangement and EMT, ultimately regulating tumor progression.^28^ Furthermore, TRIM21 can induce aberrant IL-6 expression by modulating the TRIB2-MAPK signaling axis, leading to an imbalance in the Th1/Th2 ratio of T lymphocytes.^29^ Notably, based on the recent research, TRIM21 can specifically regulate the phosphorylation of ERK1/2 through catalyzing K27-linked polyubiquitination of ERK1/2, which potentiates the interaction between ERK1/2 and MEK1/2. This enhanced interaction promotes ERK1/2 activation, ultimately driving cell proliferation. Paradoxically, overexpression of TRIM21 triggers a negative feedback loop of ERK1/2 signaling, resulting in the suppression of *p*-ERK1/2 levels and inhibition of cellular proliferation.^30^

Given the Golgi localization of CASP, we hypothesize that CASP functions not merely by reducing global TRIM21 levels, but by creating a local TRIM21-depleted zone at the Golgi. This spatial clearance could alleviate inhibitory signals on nascent vesicles transporting oncogenic cargoes (e.g., integrins), generating compartmentalized signaling pulses essential for invasion-a model consistent with Golgi matrix scaffolding roles^31^^,^^32^ that warrants future high-resolution imaging studies. In the present study, CASP is shown to regulate overall *p*-ERK levels. Nonetheless, previous work has demonstrated that Golgi-associated scaffold proteins, such as Sef, can spatially engage and modulate MEK/ERK signaling to prevent ERK nuclear translocation and retain it within the cytoplasm.^33^ Whether CASP similarly exerts spatial control over *p*-ERK activation, and which pro-tumorigenic substrates might be phosphorylated by a locally confined ERK pool as it encounters cargos at the Golgi, remain to be elucidated.

In conclusion, our study provides mechanistic insights into the functional role of CASP, an alternative splicing product of the *CUX1* gene that is distinct from CDPs. By integrating proteomic data with TCGA and GEO, and validating findings through IHC, we confirmed the ectopic expression of CASP in CRC. Further analysis revealed that dysregulated CASP expression profoundly influenced the malignant behavior of tumor cells. Mechanistically, we demonstrate that CASP interacts with TRIM21 to promote its ubiquitination and degradation, and this negative regulation of TRIM21 drives tumor progression and metastasis by activating the MAPK signaling pathway.

### Limitations of the study

Despite these findings, our study has several limitations. First, large-scale cohort analyses assessing ectopic CASP expression in CRC remain lacking. Second, we do not fully delineate the biochemical mechanism by which CASP enhances TRIM21 autoubiquitination. Future studies employing reconstituted E3 ligase assays and linkage-specific ubiquitination mapping will be essential to determine whether CASP directly modulates TRIM21’s enzymatic activity or facilitates its self-assembly. In addition, the mechanisms by which CASP promotes tumor progression warrants further investigation. Specifically, how does the CASP-TRIM21 complex activate downstream MAPK signaling? What role does this complex play in other tumor types? Finally, as a Golgi-localized coiled-coil protein, does CASP influence vesicle trafficking in CRC, as other proteins in this class? These questions remain to be addressed.

## Resource availability

### Lead contact

Further information and requests for resources and reagents should be directed to and will be fulfilled by the lead contact, Ting Chen (tchen2013@zju.edu.cn).

### Materials availability

This study did not generate new unique materials or reagents.

### Data and code availability

• Data reported in this paper will be shared by the lead contact upon request. Raw Western blot images were deposited on Mendeley Data: https://doi.org/10.17632/6mzk48brxt.1.
• This paper does not report original code.
• Any additional information required to reanalyze the data reported in this paper is available from the lead contact upon request.

## Acknowledgments

This work was supported by Zhejiang Provincial Clinical Research Center for CANCER (2022E50008 and 2024ZY01056), Fundamental Research Funds for the Central Universities (226-2024-00062) and 10.13039/501100001809National Natural Science Foundation of China (82173220 and 82403888). The graphical abstract was created with BiorRender (https://www.biorender.com/).

## Author contributions

S.Z., Q.W., and T.C. designed and supervised the study. B.Z. and W.H. collected bioinformatics data and performed bioinformatics analysis. B.Z., W.D., K.X., and L.L. developed the methodology. B.Z. and T.C analyze and interpreted the data. B.Z., T.C., and Q.W. wrote and reviewed the manuscript.

## Declaration of interests

The authors declare no competing interests.

## Declaration of generative AI and AI-assisted technologies in the writing process

During the preparation of this work, the authors used ChatGPT to enhance the language and readability. After using this service, the authors reviewed and edited the content as needed and take full responsibility for the content of the publication.

## STAR★Methods

### Key resources table

**Table:** 

REAGENT or RESOURCE	SOURCE	IDENTIFIER
**Antibodies**		

Anti-CASP rabbit polyclonal	Proteintech	Cat# 11733-1-AP; RRID: AB_2086995
Anti-β-actin mouse monoclonal	Huabio	Cat# M1210-2; RRID: AB_3073045
Anti-GAPDH mouse monoclonal	Proteintech	Cat# 60004-1-Ig; RRID: AB_2107436
Anti-c-Myc mouse monoclonal	Proteintech	Cat# 67447-1-Ig; RRID: AB_2882681
Anti-Cyclin D1 rabbit monoclonal	Cell Signaling Technology	Cat# 55506; RRID: AB_2827374
Anti-p21 rabbit monoclonal	Cell Signaling Technology	Cat# 2947; RRID: AB_823586
Anti-N-cadherin rabbit polyclonal	Proteintech	Cat# 22018-1-AP; RRID: AB_2813891
Anti-Vimentin rabbit polyclonal	Proteintech	Cat# 10366-1-AP; RRID: AB_2273020
Anti-MMP-9 rabbit polyclonal	Proteintech	Cat# 10375-2-AP; RRID: AB_10897178
Anti-β-catenin rabbit polyclonal	Huabio	Cat# EM0306; RRID: AB_3068732
Anti-Flag mouse monoclonal	Sigma Aldrich	Cat# F3165; RRID: AB_259529
Anti-His-Tag mouse monoclonal	Proteintech	Cat# 66005-1-Ig; RRID: AB_11232599
Anti-HA-Tag rabbit monoclonal	Cell Signaling Technology	Cat# 3724; RRID: AB_1549585
Alexa Fluor 488 donkey anti-rabbit IgG	Abcam	Cat# ab150073; RRID: AB_2636877
Alexa Fluor 647 anti-GM130	Abcam	Cat# ab195303; RRID: AB_2889278
HRP Conjugated Goat anti-Rabbit IgG polyclonal Antibody	Huabio	Cat# HA1001; RRID: AB_2819166
HRP Conjugated Goat anti-Mouse IgG polyclonal Antibody	Huabio	Cat# HA1006; RRID: AB_2819167

**Biological samples**		

CRC and normal tissues	Zhejiang University Biobank	N/A
Colon cancer TMAs	Shanghai OUTDO BIOTECH	HCol-Ade060CS-01; HColA160CS01

**Chemicals, peptides, and recombinant proteins**		

RIPA buffer	Boster	Cat# AR0102
IP lysis buffer	Thermo Fisher Scientific	Cat# 87787
PVDF membrane	Millipore	Cat# IPVH00010
Enhanced Chemiluminescence (ECL) Reagent	Boster	Cat# AR1172
Matrigel Matrix	Corning	Cat# 356234
Lipofectamine 3000 Transfection Reagent	Invitrogen	Cat# L3000015
FLAG M2 Magnetic Beads	Sigma Aldrich	Cat# M8823
Protein A/G Magnetic Beads	MedChemExpress	Cat# HY-K0202
Anti-His Beads	Biolinkedin	Cat# L-1015
Ni-NTA Magnetic Agarose Beads	MedChemExpress	Cat# HY-K0241
DAPI	Beyotime	Cat# P0131

**Critical commercial assays**		

Cell Cycle Staining Kit	Multi Sciences	Cat# CCS012
SYBR Green qPCR Kit	Accurate Biotechnology	Cat# AG11701
CCK-8 kit	APExBIO	Cat# K1018
5×PrimeScript RT Master Mix	Takara	Cat# RR036Q
RNA-Quick Purification Kit	ES science	Cat# RN001

**Deposited data**		

TCGA CRC RNA-seq	TCGA	https://www.cancer.gov/tcga
GEO datasets	NCBI GEO	GSE41258; GSE14333; GSE39582
Raw Western blot images	This paper	https://doi.org/10.17632/6mzk48brxt.1

**Experimental models: Cell lines**		

DLD-1	ATCC	CCL-221
LoVo	ATCC	CCL-229
HCT116	ATCC	CCL-247
HT-29	ATCC	HTB-38
293T	ATCC	CRL-3216

**Experimental models: Organisms/strains**		

Mouse: BALB/c nude, female, 4–6 weeks old	Shanghai SLAC Laboratory Animal CO. LTD	N/A
Mouse: NSG mice, male, 6–8 weeks old	Shanghai Model Organisms Center, Inc	Cat# NM-NSG-001

**Oligonucleotides**		

CASP shRNA sequences	This paper	Table S4
TRIM21 siRNA	This paper	N/A
qPCR primers for CASP, CUX1, ACTB	This paper	N/A

**Software and algorithms**		

GraphPad Prism 9	GraphPad Software	https://www.graphpad.com
ImageJ	NIH	https://imagej.nih.gov/ij/
CytExpert	Beckman Coulter	https://www.beckman.com/flow-cytometry/software/cytexpert

### Experimental model and study participant details

#### Clinical samples

Six pairs of freshly frozen CRC tissues and matched normal surgical margin tissues were obtained from the Biobank of the Cancer Institute, Zhejiang University, for immunoblotting. Written informed consent was obtained from all patients or their legally authorized representatives prior to sample collection. The use of human biological materials was approved by the Ethics Committee of the Second Affiliated Hospital, Zhejiang University School of Medicine (Approval No. 2020-666) and conducted in accordance with applicable institutional and national regulations. Patient clinical characteristics are summarized in Table S1.

Colon cancer tissue microarrays (TMAs) were purchased from Shanghai OUTDO BIOTECH CO., LTD for IHC. Two TMAs were used: HCol-Ade060CS-01 (four normal colon tissues and 28 paired colon cancer/adjacent tissues) and HColA160CS01 (80 paired colon cancer/adjacent tissues). After excluding cases with missing tissue cores, 28 CRC samples (27 with matched adjacent tissue) from HCol-Ade060CS-01 and 79 CRC samples (70 with matched adjacent tissue) from HColA160CS01 were retained for all downstream analyses. Written informed consent was obtained from all patients or their legally authorized representatives. Use of these TMAs was approved by the provider’s ethics committee (Approval Nos. SHYJS-CP-1410002 and SHYJS-CP-1701008), and conducted in accordance with applicable regulatory standards. Patient clinical characteristics are provided in Tables S2 and S3.

#### Cell culture and cell lines

The DLD-1, LoVo, HCT116, HT-29, and 293T cell lines were cultured with RPMI 1640 or DMEM medium (Gibco, Thermo Fisher Scientific) containing 10% fetal bovine serum (FBS, BI Industry). The cells were incubated at 37°C with 5% CO_2_.

#### Animal models

All animal experiments were approved by the Animal Ethics Committee of Zhejiang Chinese Medical University and conducted in accordance with the institution’s Animal Study Guidelines (Ethical No. 20230123-05) and relevant regulatory requirements. For the subcutaneous xenograft model, 4–6-week-old female BALB/c nude mice were used. For the metastatic model, 6–8-week-old male NSG mice were used.

### Method details

#### Bioinformatic analysis

All Level 3 CRC RNASeqV2 mRNA expression profiles and clinical data of CRC patients were retrieved from TCGA. Genes with expression levels below 1 in more than 50% of the samples were excluded. Raw CEL files for GSE41258, GSE14333, and GSE39582 were downloaded from the Gene Expression Omnibus (https://www.ncbi.nlm.nih.gov/geo/).

Survival differences between groups with high or low CASP expression were assessed using the Kaplan–Meier method and analyzed with the log-rank test via the *survfit* and *survdiff* functions in the R *survival* package.^34^

The expression mass data was re-analyzed by updated version of DIA-NN software. Differentially expressed proteins (DEPs) between subgroups were selected according to *P* value < 0.05 and fold change > 2 based on Student's *t* test.

Immune cell infiltration levels in CRC samples were evaluated using the CIBERSORT and xCELL algorithms. Standardized gene expression data were obtained from GEO datasets. The CIBERSORT algorithm, which employs a linear support vector regression to deconvolve transcriptome data, was applied to estimate the relative proportions of 22 human immune cell phenotypes.^35^ Concurrently, the xCELL algorithm was used to calculate enrichment scores for 64 immune and stromal cell types based on gene signature enrichment analysis.^36^

#### Immunohistochemistry (IHC)

An anti-CASP rabbit antibody (1:3000, 11733-1-AP, Proteintech) was used for IHC staining. The expression of CASP was evaluated by the immune reactive score (IRS), calculated as IRS = staining intensity × percentage of positive cells. The percentage of positive cells was graded as follows: 0 (negative), 1 (<10%), 2 (10–50%), 3 (51–80%), and 4 (>80%). The intensity was graded as follows: 0 (no staining), 1 (weakly positive, light yellow), 2 (moderately positive, brown-yellow), and 3 (strongly positive, brown) (Figure S2C). CASP expression was classified as low (IRS ≤ 4) or high (IRS ≥ 9).

#### Stable cell lines with CASP overexpression and knockdown construction

Specific shRNAs targeting CASP and a scramble control sequence were designed (Table S1) and cloned into the pLKO vector. CASP cDNA (NM_001913.5), amplified from colon cancer cells, was cloned into pCDH-EF1-Puro or pcDNA3.1-3×FLAG expression vectors.

CASP-overexpression, CASP-shRNA, and control plasmids were co-transfected with psPAX2 and PMD2.G plasmids into 293T cells. After 48 hours, the supernatant was collected and filtered through a 0.45 μm filter for subsequent infections. For viral transduction, 5×10^4^ cells were seeded in 12-well plates and incubated with 1 mL lentiviral solution containing 8-12 μg/mL polybrene. Following 48-72 hours of transduction, cells were selected with 2 μg/mL puromycin until complete elimination of uninfected cells.

#### Western blot

Proteins were extracted using RIPA buffer (AR0102, Boster), separated on 10% SDS-PAGE gels, and transferred to PVDF membranes (Millipore). The following primary antibodies were used: β-actin (1:5000, M1210-2, Huabio), GAPDH (1:50000, 60004-1-Ig, Proteintech), CASP (1:2000, 11733-1-AP, Proteintech), c-Myc (1:5000, 67447-1-Ig, Proteintech), Cyclin D1 (1:1000, 55506, Cell Signaling Technology), p21 (1:1000, 2947, Cell Signaling Technology), N-cadherin (1:5000, 22018-1-AP, Proteintech), Vimentin (1:5000, 10366-1-AP, Proteintech), MMP-9 (1:1000, 10375-2-AP, Proteintech) and β-catenin (1:1000, EM0306, Huabio). Immunoblots were probed with peroxidase-conjugated secondary antibodies (1:5000, HA1001, HA1006, Huabio) and visualized using enhanced chemiluminescence detection reagent (ECL, AR1172, Boster).

#### Immunofluorescence

CRC cells were seeded in confocal dishes and cultured for 24 hours. The cells were then fixed with cold 4% paraformaldehyde for 15 minutes at room temperature (RT) and permeabilized with 0.1% Triton X-100 for 10 minutes. Blocking was performed using Blocking Buffer (P0260, Beyotime) supplemented with 0.1% Triton X-100 for 60 minutes. Cells were then incubated overnight at 4°C with primary antibodies against CASP (1:200, 11733-1-AP, Proteintech) or β-catenin (1:200, EM0306, Huabio). After washing, cells were incubated with Alexa Fluor 488-conjugated goat anti-mouse IgG (1:300, ab150073, Abcam) for 2 hours at RT. Following PBS washes, cells were incubated with Alexa Fluor® 647 Anti-GM130 (1:1000, ab195303, Abcam) for 2 hours at RT and washed off with PBS and counterstained with DAPI (P0131, Beyotime) for 10 min. Images were acquired using a LSM900 confocal microscope (Zeiss, Germany).

#### RNA isolation and quantitative reverse transcription-polymerase chain reaction (qRT-PCR)

Total RNA was extracted from CRC cells using RNA-Quick Purification Kit (RN001, ES science, Shanghai. China) following the manufacturer’s protocol. The 5×PrimeScript RT Master Mix (RR036Q, Takara) was used for reverse transcription. SYBR Green qPCR Kit (AG11701, Accurate Biotechnology, Hunan, China) was applied for RT-qPCR via Applied Biosystems 7500 Fast Real-Time PCR System. β-ACTIN was used as the loading control. The 2 − ^ΔΔCt^ method was used for relative expression calculations. Primer sequences specific to each gene were as follows:

CASP forward: 5′-GGAGGTGCTGTTGCTGGAGAAG-3′,

CASP reverse: 5′-CCGCTGGATGGACTGAATGATGCT-3′,

p200 CUX1 forward: 5′-CCAGAGCCTGAACAGACTATTT-3′,

p200 CUX1 reverse: 5′-CTTTAAGGCAGGGTCGAGGGCA-3′,

β-ACTIN forward: 5′-AGCACTGTGTTGGCGTACAG3′,

β-ACTIN reverse: 5′-TCCCTGGAGAAGAGCTACG.

#### Cell viability assessment and cell cycle assay

The proliferative ability of CRC cells was assessed using the Cell Counting Kit-8 (CCK-8, K1018, APExBIO). CRC cells (1-1.5 × 10^3^ per well) were seeded into 96-well plates. After allowing cells to adhere and recover, 10 μL of CCK-8 reagent was added to 100 μL of culture medium daily for 7 consecutive days. Plates were incubated in the dark at 37°C for 90 minutes, and absorbance at 450 nm was measured using an ELISA reader.

For cell cycle analysis, the Cell Cycle Staining Kit (CCS012, Multi Sciences Biotech) was used following the manufacturer’s protocol. Stained cells were analyzed using a DxFLEX flow cytometer (Beckman), and results were processed with CytExpert software.

#### Colony formation assay

A total of 500-800 CRC cells were seeded into a 6-well plate. After 10 days, cell clones were formed, fixed with 4% paraformaldehyde for 15-30 minutes, and washed with PBS. Crystal violet staining solution was then added to each well and incubated at room temperature for 15-30 minutes. The cells were washed with PBS several times until the plate background was clear. Once dried, the colonies were photographed.

#### Cell migration and invasion assays

A Transwell assay with 8 μm pore chambers (3422, Corning) was used to evaluate the metastatic potential of CRC cells. Chambers were pre-coated with Matrigel (356234, Corning) to mimic the extracellular matrix. A 200 μL FBS-free suspension containing 6 × 10^4^ (migratory) or 8 × 10^4^ (invasive) DLD-1 cells, or 1 × 10^5^ LoVo/HCT116 cells, was added to the upper chamber, while 800 μL of culture medium with 20% FBS was added to the lower chamber. After 24 hours (DLD-1) or 48 hours (LoVo, HCT116), the upper chamber was washed with PBS, fixed with 4% paraformaldehyde for 15 minutes at RT, and stained with crystal violet for 15-30 minutes. Non-migrated cells on the upper membrane surface were removed with a cotton swab. Migrating and invading cells were visualized using an inverted optical microscope (Axio Observer 3, Zeiss) and quantified with ImageJ software.

#### Co-immunoprecipitation (Co-IP) assay

Following 48-hour transfection with the indicated plasmids, cells were lysed using IP lysis buffer (87787, Thermo Fisher Scientific). The lysates were then subjected to immunoprecipitation with FLAG M2 Magnetic Beads (M8823, Sigma Aldrich), Anti-His Beads (L-1015, Biolinkedin), or Ni-NTA Magnetic Agarose Beads (HY-K0241, MedChemExpress), strictly following the respective manufacturers’ protocols.

For endogenous co-immunoprecipitation (Co-IP), DLD-1 or LoVo cells were collected and lysed. The cell lysates were incubated overnight at 4°C with Protein A/G beads (HY-K0202, MedChemExpress) that had been pre-coupled with either a CASP antibody or control IgG antibody for 30 minutes at room temperature. Thereafter, the bead-bound proteins were washed with IgG Elution Buffer (21028, Thermo Fisher Scientific) and neutralized with Tris-HCl (pH 8.8). The eluted proteins were finally analyzed by western blotting (WB).

Identification of interacting proteins was carried out via liquid chromatography–tandem mass spectrometry (LC-MS/MS) at Oebiotech (Shanghai, China).

#### siRNA knockdown

DLD-1 cells were seeded into 12-well plates. When the cells reached 50–60% confluence, siRNA (GenePharma, Shanghai, China) was transfected into the cells using Lipofectamine 3000 (Invitrogen) following the manufacturer’s instructions. After 48 hours of incubation, the cells were harvested for subsequent experiments. The sequences of TRIM21 siRNA were as follows: sense: 5′-GCAUGGUCUCCUUCUACAATT-3′ and antisense: 5′-UUGUAGAAGGAGACCAUGCTT-3′.

#### Turnover assay

CASP-KD DLD-1 cells and the respective control cells were seeded in 12-well plates. Following a 24-hour incubation, the culture medium was replaced with one containing CHX, 60 (μg/ml) to inhibit protein synthesis. Cells were lysed at the indicated time points after CHX exposure. The resulting protein extracts were subjected to WB analysis to determine the abundance of the target protein over time.

#### Ubiquitination assay

293T cells were co-transfected with plasmids encoding CASP-shRNA or negative control, TRIM21-His and HA-Ub plasmids for 72 hours. Six hours prior to harvest, the cells were treated with 20 μM MG132 to inhibit proteasomal degradation. Cell lysates were incubated with Ni-NTA agarose to pull down His-tagged proteins. After extensive washing, the bound proteins were eluted by boiling in 2× SDS loading buffer for 5 minutes. Ubiquitination levels were subsequently analyzed by WB using the indicated antibodies.

#### Animal experiments

For the subcutaneous tumor model, a total of 5 × 10^6^ CRC cells in 100 μL were injected into the right flank of nude mice. Mice were randomly divided into two groups: Scramble (n=5) and shCASP-1 (n=5). Tumor sizes were measured every 4 days, and tumor volume was calculated using the formula: Volume = ½ × length × width^2^. After 45 days (DLD-1) or 53 days (LoVo), mice were euthanized, and tumors were excised and weighed.

For the metastatic tumor model, a total of 2 × 10^6^ LoVo cells in 200 μL were injected into the tail vein of NSG mice. Mice were randomly divided into two groups: Scramble (n=5) and shCASP-1 (n=5). *In vivo* bioluminescence imaging was performed on the day of injection and every 5-12 days thereafter to monitor metastatic tumor formation. On day 34, mice were sacrificed, and their lungs and livers were dissected, imaged *ex vivo*, and fixed in 4% paraformaldehyde.

### Quantification and statistical analysis

All statistical analyses and graphical representations were performed using GraphPad Prism v9.0. Quantitative data were presented as means ± SD. For comparisons between two groups, unpaired or paired Student’s t-tests were employed, depending on the experimental design. A Chi-square test was performed to examine the association between categorical variables. Pearson’s correlation coefficient was calculated to determine the strength of associations. Statistical significance was defined as a two-sided *P* value < 0.05 (∗*P* < 0.05, ∗∗*P* < 0.01, ∗∗∗*P* < 0.001, ∗∗∗∗*P* < 0.0001, *ns* = no significant difference).
