# Supplementary material for: Functional characterization of CASP, a CUX1 isoform, reveals its tumor-promoting role in colorectal cancer via TRIM21-mediated signaling
Source: iScience. 2026 Jan 29;29(2):114783. doi: 10.1016/j.isci.2026.114783 (PMC12915277; doi:10.1016/j.isci.2026.114783)
Supplement: Document S1. Figures S1–S6 and Tables S1–S4 [file mmc1.pdf]

iScience, Volume *29*

Supplemental information

Functional characterization of CASP, a *CUX1* isoform, reveals its tumor-promoting role in colorectal cancer via TRIM21-mediated signaling

Biting Zhou, Wangxiong Hu, Wei Dai, Kailun Xu, Lihong Liu, Shu Zheng, Qichun Wei, and Ting Chen

Figure S1

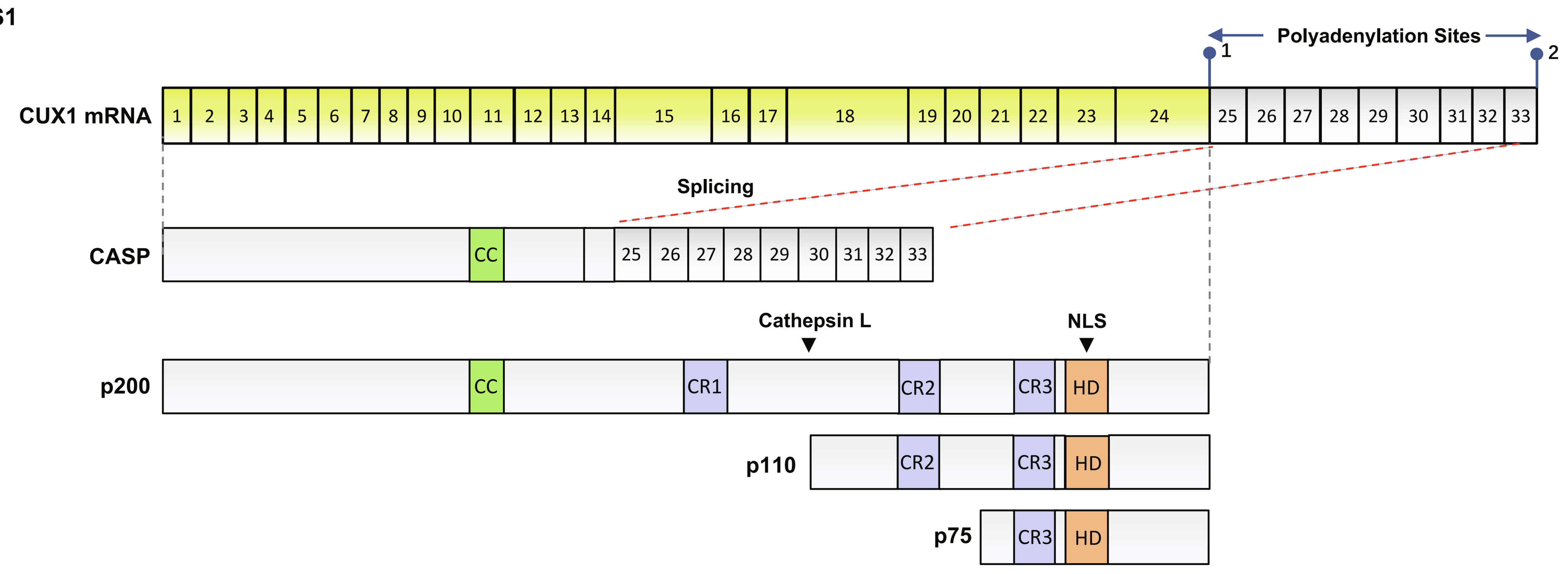

Figure S1. Structure of CUX1 mRNA and its major protein isoforms.

CC: coiled-coil, CR: CUT repeat, HD: homeodomain, NLS: nuclear localization signal.

Figure S2

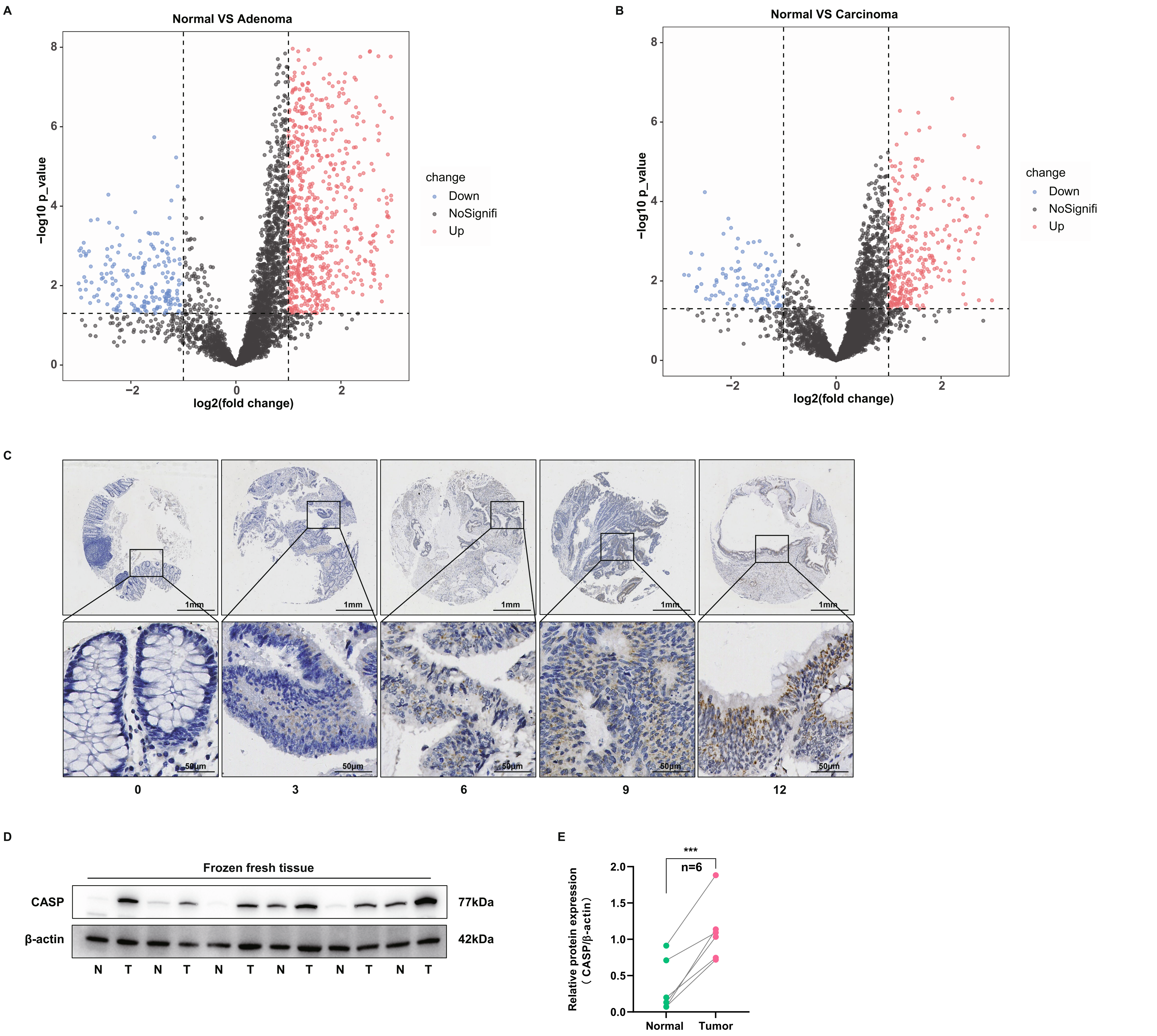

Figure S2. Proteomic screening identifies dysregulated proteins in CRC progression and validation of CASP expression and scoring (related to Figure 1).

(A) Differentially expressed proteins between normal tissues vs. adenomas.

(B) Differentially expressed proteins between normal tissues vs. CRC.

(C) Illustration of the CASP IHC scoring.

(D) WB analysis of CASP expression in six matched frozen CRC tissues and normal margins.

(E) The band intensities from WB analysis were quantified using ImageJ software and target protein bands was normalized to the corresponding control (β-actin or GAPDH) to account for variations in sample loading.

Data are expressed as mean ± SD. Statistical analyses were performed on GraphPad Prism. (E) Paired *t* test, ****P* < 0.001.

Figure S3

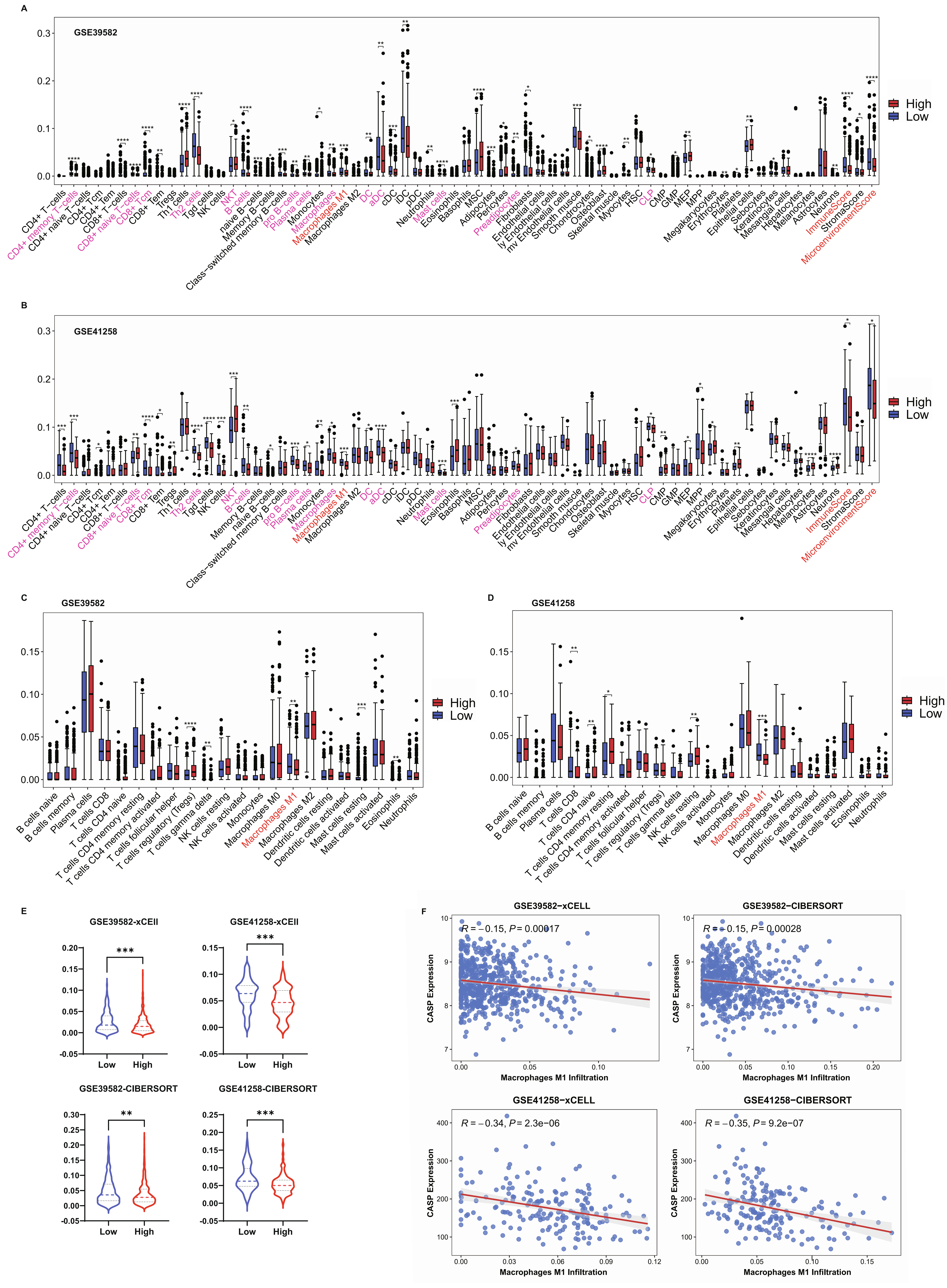

Figure S3. Comparative analysis of tumor immune infiltration based on CASP expression.

(A and B) Differential enrichment scores of 64 cell types, along with Immune, Stromal, and Microenvironment scores, between CASP-high and CASP-low groups in the (A) GSE39582 and (B) GSE41258 datasets, as assessed by xCELL.

(C and D) Differences in the relative proportions of 22 immune cell types between CASP-high and CASP-low groups in the (C) GSE39582 and (D) GSE41258 datasets, as determined by CIBERSORT.

(E) Violin plot depicting the differential infiltration levels of M1 macrophages between CASP-high and CASP-low groups.

(F) Scatter plot showing a significant negative Pearson correlation between the infiltration level of M1 macrophages and CASP expression levels across the two datasets. Data are expressed as mean ± SD. Statistical analyses were performed on GraphPad Prism. (A-E) Unpaired *t* test. (F) Pearson's correlation analysis. $^{*}P < 0.05$, $^{**}P < 0.01$, $^{***}P < 0.001$, $^{****}P < 0.0001$.

Figure S4

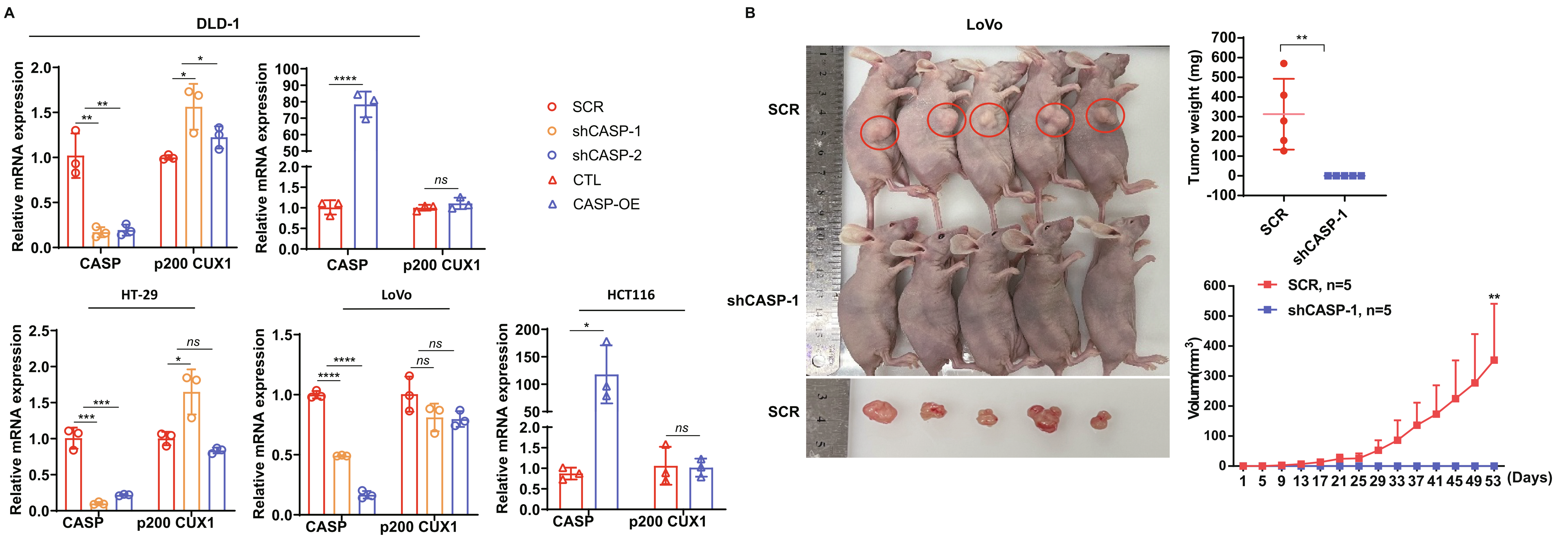

Figure S4. CASP expression levels in CRC cells and its impact on tumor growth *in vivo* (related to Figure 2).
(A) Quantification analysis of relative mRNA level of CASP and P200 CUX1 of CRC cell lines.
(B) Subcutaneous tumor formation in nude mice (n=5/group) with CASP-KD cells; tumors derived from LoVo cells (left) and tumor growth curve of control (scramble) and CASP-KD LoVo cells in xenograft model (right).
Data are expressed as mean ± SD. Statistical analyses were performed on GraphPad Prism. (A-B) Unpaired *t* test. **P* < 0.05, ***P* < 0.01, ****P* < 0.001, *****P* < 0.0001, *ns* = no significant difference.

Figure S5

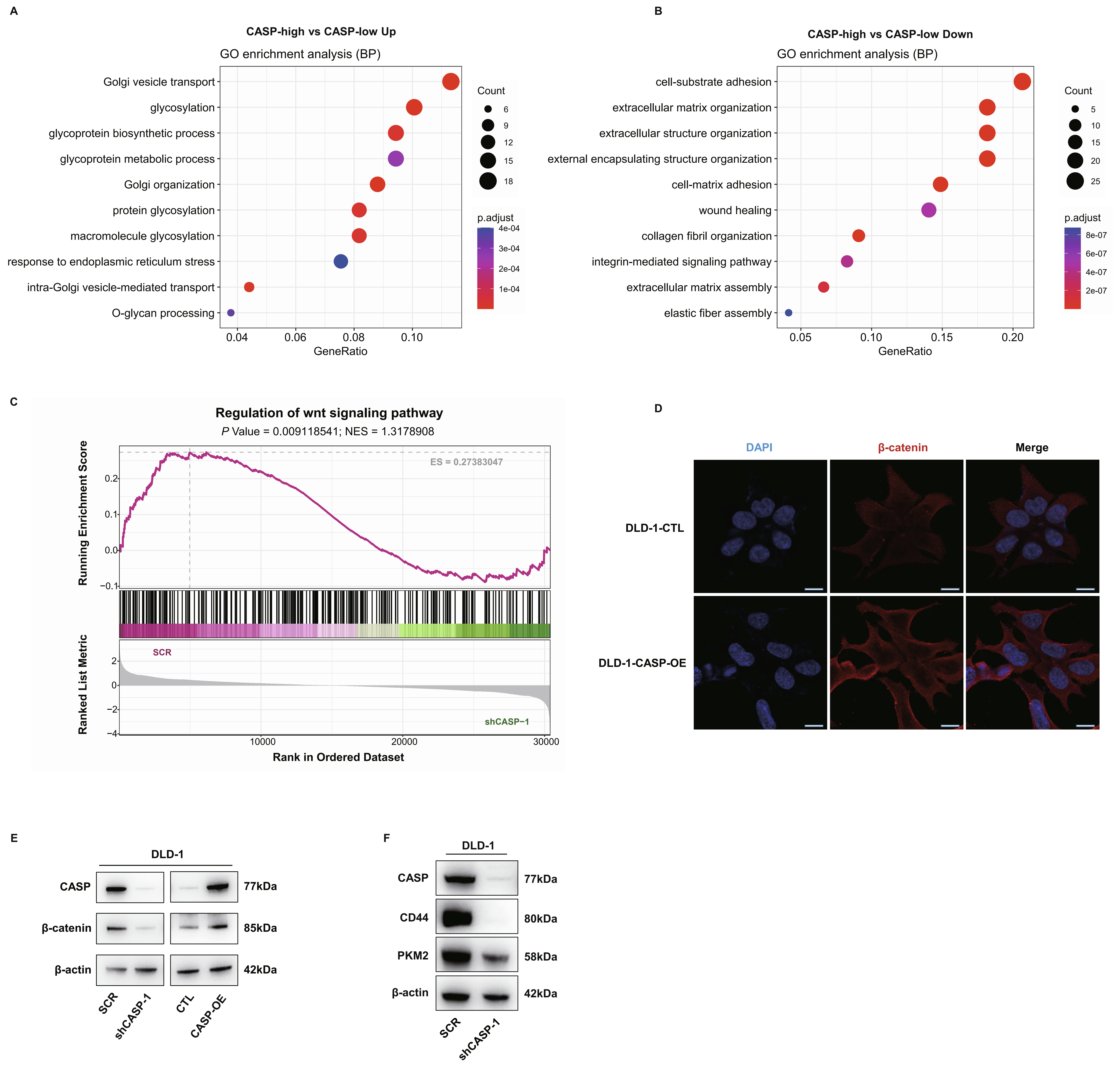

Figure S5. Functional enrichment analyses and experimental validation of CASP-regulated Wnt/β-catenin signaling activation and downstream target gene expression (related to Figure 4).
(A and B) Bubble plots of GO analysis of up-regulated proteins (A) and down-regulated proteins (B) between CASP-high and CASP-low group.
(C) GSEA analysis shows the aberrant regulation of Wnt signaling pathway.
(D and E) Immunofluorescence (D) and WB (E) show the accumulation of β-catenin in CASP-OE DLD-1 cells and the reduction in CASP-KD DLD-1 cells, scale bar: 10 μm.
(F) WB analysis of CD44 and PKM2 in scramble and CASP-KD DLD-1 cells.

Figure S6

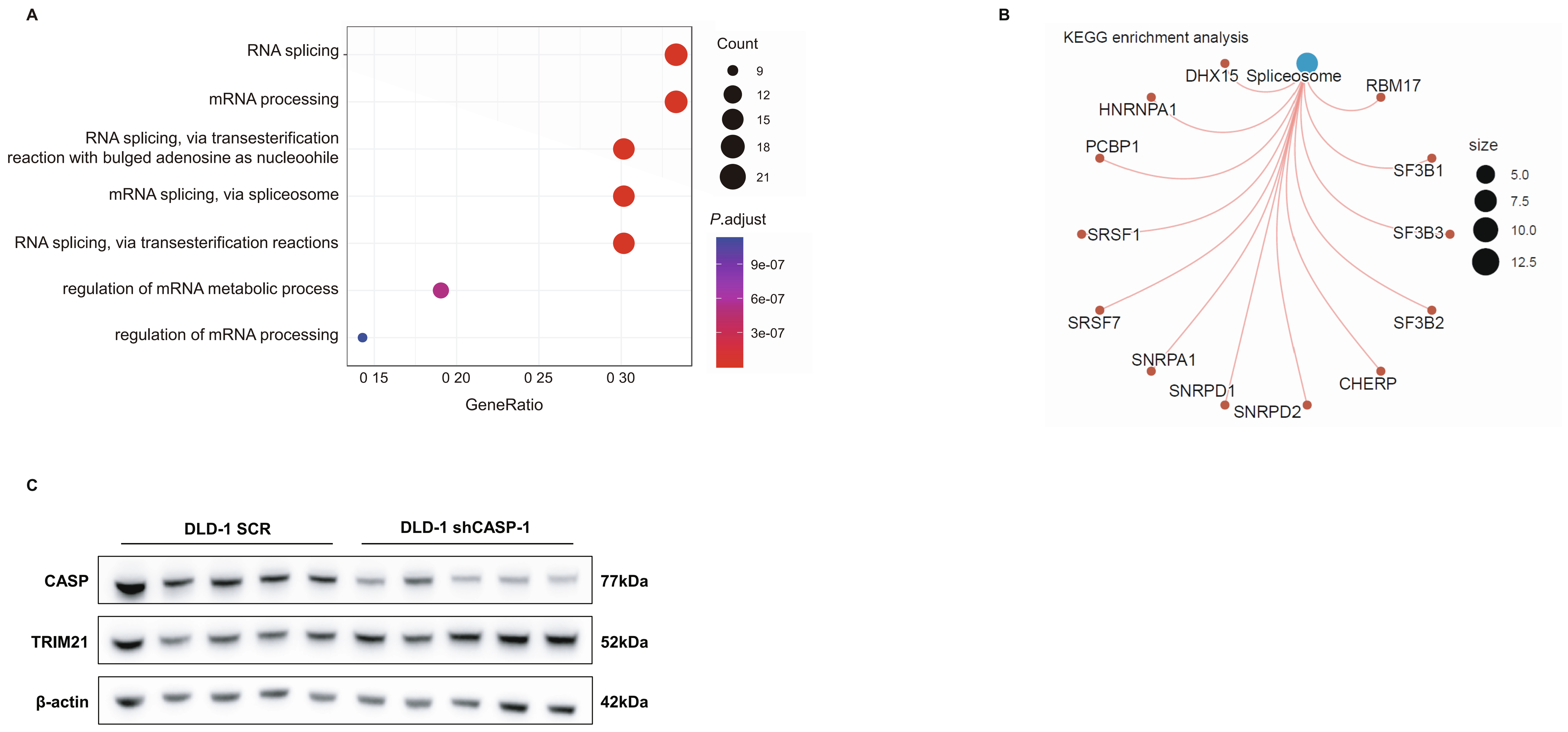

Figure S6. Co-IP-based proteomic profiling, functional enrichment analysis, and *in vivo* validation of TRIM21 as a CASP interactor (related to Figure 5).
(A-B) GO enrichment (A) and KEGG pathway enrichment (B) analysis of proteins identified by Co-IP in CASP-OE DLD-1 cells.
(C) WB analysis of TRIM21 and CASP expression in protein extracts derived from subcutaneous xenograft tumors grown in nude mice.

Table S1. Clinical characteristics of six CRC patients with frozen tissues.

Sample ID	Gender	Age (years)	TNM stage	Differentiation degree	Tumor location
T1	Female	69	I	Moderate	Transverse
T2	Female	65	IIA	Poor	Right
T3	Male	68	IIA	Moderate	Right
T4	Female	65	IVA	Moderate	Left
T5	Female	70	IIA	Poor	Right
T6	Male	57	IIIA	Moderate	Rectum

Table S4. CASP shRNAs and Scramble control sequence.

	Forward (5'-3')	reverse (5'-3')
**shCASP-1**	CCGGAGATCCCAGAGCCC	AATTCAAAAAAGATCCCAG
	ATCAAAGCTCGAGCTTTGA	AGCCCATCAAAGCTCGAGC
	TGGGCTCTGGGATCTTTTT	TTTGATGGGCTCTGGGATC
	TG	T
**shCASP-2**	CGGCTTCTTCTACACACTG	CAAAAACGGCTTCTTCTAC
	TTCTCGAGAACAGTGTGTA	ACACTGTTCTCGAGAACAG
	GAAGAAGCCGTTTTTG	TGTGTAGAAGAAGCCG
**Scramble**	CCGGCAACAAGATGAAGAG	AATTAAAAAACAACAAGATG
	CACCAACTCGAGTTGGTGC	AAGAGCACCAACTCGAGTT
	TCTTCATCTTGTTGTTTTTT	GGTGCTCTTCATCTTGTTG

Table S2. Clinical characteristics of colon cancer patients from TMA HCol-Ade060CS-01.

Characteristics	Category	Number (%)
**Gender**	Male	20 (71.43)
	Female	8 (28.57)
**Age***	>60 years	15 (55.56)
	≤60 years	12 (44.44)
**Tumor location**	Left	13 (46.43)
	Right	11 (39.29)
	Transverse	4 (14.29)
**Differentiation degree**	Well	1 (3.57)
	Moderate	24 (85.71)
	Poor	3 (10.71)
**TNM stage**	I	6 (21.43)
	II	8 (28.57)
	III	7 (25.00)
	IV	7 (25.00)
**Vascular invasion**	Present	3 (10.71)
	Absent	25 (89.29)
**Perineural invasion**	Present	2 (7.14)
	Absent	26 (92.86)

**Age was not available for one patient.*

Table S3. Clinical characteristics of colon cancer patients from TMA HColA160CS01.

Characteristics	Category	Number (%)
**Gender**	Male	43 (54.43)
	Female	36 (45.57)
**Age**	>60 years	52 (65.82)
	≤60 years	27 (34.18)
**Tumor location**	Left	28 (35.44)
	Right	40 (50.63)
	Transverse	11 (13.92)
**Differentiation degree**	Moderate	61 (77.22)
	Poor	18 (22.78)
**TNM stage**	I	8（10.13）
	II	44（55.70）
	III	23（29.11）
	IV	4（5.06）
**Vascular invasion***	Present	5（6.41）
	Absent	73（93.59）
**Perineural invasion**	Present	10（12.66）
	Absent	69（87.34）
**MMR status**	Proficient	59（74.68）
	Deficient	20（25.31）

**An equivocal case of lymphovascular invasion was omitted from the final tally.*
